# History of a Successful Case of Cæsarian Operation

**Published:** 1830

**Authors:** John L. Richmond

**Affiliations:** Newtown, Hamilton county, Ohio


Art. II.—*History of a successful case of Casarean Operation.*— By John L. Richmond, M. D. of Newton, Ohio.

On the 22d of April 1827, I was called to visit a Miss E. C. in labour; on my arrival at the house, I found she had been in labour about 30 hours. Two midwives had been called, but neither of them could give any account of the case, except that "she had fits and the pains did no good."

On examination, I found that the os externum, had suffered no dilatation, and there was no fœtal tumour in the pelvis, except when the pain was on, when there was a kind of pressing down of the uterus and the contents of the pelvis. The uterus presented a smooth tumour towards the superior extremity of the vagina, which seemed only to be felt through the anterior part of the vagina, and the anterior part appeared to form an acute angle with the posterior, immediately in the hollow of the sacrum, and a little posterior to the tumour.

She lay, by spells, comparatively easy; when her pains came on, they continued for a short space of time, nearly regular or natural, but in twenty or thirty seconds were transfered to the stomach, and immediately terminated in general convulsions, which continued from three to five minutes, and were succeeded by alarming faintings, which lasted from ten to twenty minutes. The system was much exhausted, the pulse depressed, and not the least advantage had yet resulted from all she had suffered.

My first object was, to prevent the convulsions and to recruit the system; for which purpose I gave laudanum and sulphuric ether, and applied flannel wet with hot spirits to the feet. These measures produced considerable mitigation of the convulsions, but the fainting increased. I had no recourse to cordials, for these could not be obtained. I was seven miles from home, and had but few medicines with me. I spent four hours in fruitless attempts, either to recruit my patient or to ascertain the exact condition of the mother, or the presentation of the child. The vagina seemed a kind of sack, the extremity of which could easily be reached with the finger, but nothing like a uterus, could be felt, except a tumour above, which was felt through the vagina; under these circumstances, finding my patient fast sinking, I requested advice, which, however, could not be obtained, on account of high water in the Little Miami and the darkness of the night.

I informed the patient and her friends, of the only means by which I could conceive of relief; this was at once consented to as affording some hopes of life.

After doing all in my power for her preservation, and feeling myself entirely in the dark as to her situation, and finding that whatever was done, must be done soon, and feeling a deep and solemn sense of my responsibility, with only a case of common pocket instruments, about one o'clock at night, I commenced the Caesarean Section. Here I must take the liberty to digress from my subject, and relate the condition of the house, which was made of logs that were green, and put together not more than a week before. The crevices were not chinked, there was no chimney, nor chamber floor. The night was stormy and windy, insomuch, that the assitants had to hold blankets to keep the candles from being blown out. Under these circumstances it is hard to conceive of the state of my feelings, when I was convinced that the patient must die, or the operation be performed.

I commenced the operation, by making an incision through the integuments, down to the *linea alba* from the umbilicus, to within an inch and a half of the pubis. I then made a short incision through the tendon, about one third of the way from the lower extremity of the other, and introducing my finger, I found that the omentum was much in the way, as she was very fat. I introduced the blade of a crooked pair of scissors, and crowding the omentum up with my finger, cut first up and then down. During this part of the operation, the hæmorrhage was very trifling. I presume not exceeding four or five ounces.

As soon as the tension of the abdominal muscles was taken off, the convulsions subsided, and the patient became composed and tranquil. The uterus then presenting, I proceeded to divide it in the same manner as I had done the *linea alba.* I made the incision from as low down as I could, to near the fundus uteri; the incision passed immediately over the placenta. This incision produced considerable hæmorrhage, which however, soon partially subsided and I, then, divided the placenta, by making a small incision in it, and then lacerating it, which I thought would occasion less hemorrhage than to cut the whole of it. I then suffered all the blood to escape that I could, while the whole cavity of the abdomen was filled; and wiped away all I could, before trying to remove the child.

The child lay with the back presenting to the incision, the head resting on the superior strait of the pelvis; the uterus and placenta being thus divided, the contraction of the former were rapid, and the latter soon became entirely detached. As soon as the gush of blood partially subsided, I commenced my efforts to remove the child; but as it was uncommonly large, and the mother very fat, and having no assistance, I found this part of my operation more difficult than I had anticipated. My first endeavor was to raise the child sufficiently towards the stomach, to bring the head from under the pubis; but this I was unable to do, by any force which appeared to me safe to exert. I then made several vain attempts to raise the breech. After which I endeavored to pass my hand around the child, and get hold of the feet, but this the patient could not endure; and thinking the danger of the mother very great; and believing or supposing, that the child was dead from the detachment of the placenta; and considering, at all events, that a childless mother, was better than a motherless child, I determined to do all I could for the preservation of the mother. Accordingly I made a transverse incision across the back of the foetus, near the upper lumbar vertebræ, and the muscles of the back being divided, it formed an angle instead of a curve, by which means I was enabled, easily to extract it. The placenta, being entirely detached from the uterus, was at once removed, and the blood carefully wiped out of the uterus and all the surrounding parts properly cleansed.

I now determined to make, if possible, some discovery in relation to the *orificium uteri.* I accordingly passed my hand into the uterus; and, by examining carefully, I found an apperture which, to the touch, from within, did not seem to bear any resemblance to a natural orifice. I introduced the finger of the other hand, into the vagina and could not bring them into contact with each other—there seemed to be a kind of tube, leading from the uterus, to within about three fourths of an inch of the *meatus urinarius* into which I could not pass my finger at the upper extremity, to any distance, and not at all below. I then dressed the wound in the common manner, with sutures and adhesive straps, leaving about two inches of the lower extremity open.

She now lay perfectly easy and went to sleep. I kept her in one position for four days, keeping the bowels open with saline purges and injections. The loehial discharge commenced in about eight hours, and continued for five days; some discharge also occured from the open part of the incision. That part of the wound which was closed, adhered by the first intention. I suffered her to take no nourishment but weak gruel. On the seventh day, I closed the lower part of the wound; but finding, on the twelfth, that an accumulation had taken place in the cavity of the abdomen, I opened a small orifice from which a large quantity of black very offensive blood and water, was discharged. I then introduced a female catheter, and with a pint syringe, threw in three pints of warm water with a small quantity of soap in it, and drew it back with the syringe, after the manner of a stomach pump; this I repeated six successive days, when the water which was injected ceased to be coloured and the orifice was suffered to close. The patient never complained of pain during the whole course of the cure. She commenced work in twenty-four days from the operation, and in the fifth week walked a mile and back the same day.

One circumstance I cannot forbear relating. As I was syringing out the abdomen, as above mentioned, a neighboring woman, standing by my side, said to her what makes you laugh? to which she replied, because it feels so queer. I looked to her face and she was laughing.

I have made a recent examination of this patient, *per vaginam*, and the condition of the vagina remains as above described, only it is now more shallow than it was when the uterus was raised into the abdomen; the whole depth of the vagina is now only two thirds of a fingers length, the orifice, or abnomral *os tincæ*, would not be discovered by the most minute examiner, who was not apprised of its situation. The anterior coat of the vagina now feels like a kind of septum, passing obliquely upward from before backward, leaving, I think, about one and a half inches between it and the forchet. I should think, if it were possible, that it is an unnaturally situated hymen. Here is as much room for others to theorize on the physiology of conception as for me. She has been married since and lived two years with a husband, during which time she tells me that she suffered great inconvenience on account of the shallowness of the vagina, but no conception has taken place. She suffers no inconvenience from the abdominal cicatrix, it being perfectly firm.

Newton, Hamilton county, Ohio, Feb. 1830.

